# A Systematic Review of Chronic Pain in People with Schizophrenia

**DOI:** 10.1192/j.eurpsy.2025.1893

**Published:** 2025-08-26

**Authors:** S. Krug, M. Edwards

**Affiliations:** 1 KCL Institute of Psychiatry, Psychology & Neuroscience, London, United Kingdom

## Abstract

**Introduction:**

Previous research has shown chronic pain to be more prevalent in individuals with psychiatric disorders, compared to the general population.

**Objectives:**

We performed a systematic review of studies relating to chronic pain in patients with schizophrenia (PWS), to explore its cause, prevalence and presentation.

**Methods:**

Our search strategy yielded 4963 studies. Once duplicates were removed, and studies were screened according to our inclusion/exclusion criteria, 15 studies on chronic pain and quality of life (QOL) in PWS remained.

**Results:**

Our results showed that the prevalence of chronic pain in PWS was equal to, or greater than, healthy controls. Studies assessing chronic headaches specifically, found headaches to be more prevalent. Studies that compared chronic pain in PWS to individuals with other psychiatric disorders, such as depression or bipolar disorder, found PWS to have lower levels of pain. Pain intensity ranged from mild to moderate and was most frequently reported in the abdomen and head. The presence of pain was associated with anxiety, depression, psychotic symptoms, and older age. No clear links were found between chronic pain and patient gender, education, or wealth. QOL, particularly health-related QOL, was lower in patients with higher levels of pain, and such patients experienced greater functional impairment. However, when PWS performed self-assessments of QOL and health satisfaction, no difference was seen between individuals with and without pain.

**Conclusions:**

These variations in pain perception may be due to disturbances in somatosensation, with PWS being internally more preocccupied. Specifically, computational models suggest this may be due to aberrant salience, where PWS attribute meaning or value to innocuous stimuli. Understanding the link between chronic pain and schizophrenia is essential as this may contribute to premature death. Further research is required to explore the link between comorbidities as a cause of chronic pain in PWS.

**Disclosure of Interest:**

None Declared

